# Nutritional Combined Greenhouse Gas Life Cycle Analysis for Incorporating Canadian Yellow Pea into Cereal-Based Food Products

**DOI:** 10.3390/nu10040490

**Published:** 2018-04-16

**Authors:** Abhishek Chaudhary, Christopher P. F. Marinangeli, Denis Tremorin, Alexander Mathys

**Affiliations:** 1 Institute of Food, Nutrition and Health, Eidgenössische Technische Hochschule (ETH) Zürich, CH-8092 Zürich, Switzerland; alexander.mathys@hest.ethz.ch; 2 Pulse Canada, Winnipeg, MB R3M 0A5, Canada; cmarinangeli@pulsecanada.com (C.P.F.M.); dtremorin@pulsecanada.com (D.T.)

**Keywords:** peas, pulses, nutrition, nutrient density, agriculture, carbon footprint, greenhouse gas, bread, cereal, pasta

## Abstract

Incorporating low cost pulses, such as yellow peas, that are rich in nutrients and low in fertilizer requirements, into daily food items, can improve the nutritional and sustainability profile of national diets. This paper systematically characterized the effect of using Canadian grown whole yellow pea and refined wheat flours on nutritional density and carbon footprint in cereal-based food products. Canada-specific production data and the levels of 27 macro- and micronutrients were used to calculate the carbon footprint and nutrient balance score (NBS), respectively, for traditional and reformulated pan bread, breakfast cereal, and pasta. Results showed that partial replacement of refined wheat flour with yellow pea flour increased the NBS of pan bread, breakfast cereal, and pasta by 11%, 70%, and 18%, and decreased the life cycle carbon footprint (kg CO_2_ eq/kg) by 4%, 11%, and 13%, respectively. The cultivation stage of wheat and yellow peas, and the electricity used during the manufacturing stage of food production, were the hotspots in the life cycle. The nutritional and greenhouse gas (GHG) data were combined as the nutrition carbon footprint score (NCFS) (NBS/g CO_2_ per serving), a novel indicator that reflects product-level nutritional quality per unit environmental impact. Results showed that yellow pea flour increased the NCFS by 15% for pan bread, 90% for breakfast cereal, and 35% for pasta. The results and framework of this study are relevant for food industry, consumers, as well as global and national policy-makers evaluating the effect of dietary change and food reformulation on nutritional and climate change targets.

## 1. Introduction

The global food demand is projected to increase by 70% by 2050 [1]. Accordingly, providing sufficient food and adequate nutrition, while minimizing the impact of food systems on the environment for a growing population, has been identified as a formidable challenge. As pulses underpin significant environmental and nutritional benefits within the food value chain [2], increasing their consumption represents an opportunity to improve the nutritional density and sustainability of food systems worldwide.

Pulses are a subgroup of legumes that are solely harvested as a dry grain [3]. From an environmental perspective, pulses, particularly lentils and peas, have been shown to improve the sustainability of cropping systems by breaking disease cycles, and improving soil health and soil fertility, as well as improving the yield and protein content of succeeding cereal crops [4]. Importantly, pulses are well adapted to semi-arid conditions, have lower water requirements [5], and have been shown to improve soil microbial diversity [6]. Given their ability to fix nitrogen and reduce nitrogen fertilizer requirements, yellow peas and lentils have been characterized as some of the lowest carbon footprint foods available [7].

As a significant source of macro- and micronutrients, including protein, fiber, folate, iron, potassium, and magnesium, pulses are included in dietary guidelines and cultural dietary patterns across regions as nutrient-dense foods [8]. Observational studies from Canada and the United States demonstrate that, compared to non-consumers, pulse consumers demonstrated greater macro- and micronutrient density of diets on any given day [9,10]. Enhanced use of pulses as ingredients could be a viable strategy to help improve dietary quality and concurrently decrease the carbon footprint of agriculture and food systems.

Canada is the world’s largest producer of yellow peas and, relative to production, dry yellow peas remain the most underutilized pulse in North America. Moreover, yellow peas are the lowest cost pulse available. Therefore, their utilization as a flour can be a cost-effective method for bolstering the nutritional density and sustainability profile of staple processed food products commonly consumed in North America, or other regions.

Life cycle assessment (LCA) is a comprehensive framework used to assess the environmental impact of food products from the extraction of raw material through to disposal [11,12,13,14]. LCA studies have confirmed that agriculture represents a large proportion of the environmental footprint of cereal-based foods [15]. However, the environmental footprint of cereal-based foods reformulated with pulse flours, such as yellow pea, has not been assessed. Moreover, rather than traditional LCA of food items that focus only on environmental impacts, there is interest in identifying foods and diets that are both nutritionally dense and environmentally sustainable. This type of analysis demands a more nuanced comparative basis [16,17,18,19]. In other words, there is a need for quantitative indicators and frameworks that can jointly assess the nutritional as well as environmental profile of global foods.

This study assessed the nutritional and greenhouse gas outcomes of cereal-based food products, such as pan bread, breakfast cereal, and pasta, that have been reformulated with Canadian-grown yellow pea flour as a replacement for refined wheat flour. Unlike previous studies that focused on single nutritive outcomes, such as caloric or protein levels in food [16,17,18,19], the nutritional quality of the traditional (without yellow pea flour) and reformulated (with yellow pea flour) foods was analyzed by aggregating levels of 27 micro- and macronutrients (qualifying nutrients), and five nutrients of health concern (disqualifying nutrients). The life cycle carbon footprint of 1 kg and 1 serving of traditional and reformulated products was determined by accounting for all inputs during the cultivation, milling, and manufacturing stages. To minimize uncertainty, where possible, carbon footprint assessments were based on production methods and datasets corresponding to Western Canada.

## 2. Materials and Methods

### 2.1. Ingredient Composition of Food Products

Given that one or more of pan bread, breakfast cereal, and pasta represent important sources of nutrition in various regions [20], they were used to model effects of reformulation on nutrient density and sustainability. Recipes for bread, pasta, and breakfast cereals manufactured with whole yellow pea flour were from the Canadian International Grains Institute (Winnipeg, MB, Canada) in January 2017. Recipes provided by the Canadian International Grains Institute were previously tested for performance and acceptability [21,22,23], and were used as a theoretical guide for formulations used in this analysis. The baseline formulations contained a combination of whole yellow pea flour and refined wheat flour, and were characterized as “reformulated foods” in this study. In the reformulated products, the proportion of whole yellow pea flour relative to the total flour was 15% for the pan bread, 53% for the breakfast cereal, and 30% for the pasta. Foods characterized as “traditional” formulations assumed the same proportions of ingredients where whole yellow flour was fully replaced by refined wheat flour (i.e., 0% yellow pea flour). A list of ingredients used in each food is listed in Table 1.

The type of refined wheat flour used by each product formulation varied in protein levels and reflects the type of wheat flour typically used to produce corresponding foods (Table 1). Calcium sulfate was assumed to be used as the dough conditioner for traditional and reformulated pan bread. Reformulated recipes provided by Canadian International Grains Institute included gluten as an ingredient in the pan bread, and cornmeal and pea hull flour in the breakfast cereal; these ingredients were removed for the present analysis. For the breakfast cereal, the cornmeal and pea hull flour were replaced with refined wheat flour. With the exception of moisture, it was assumed that the energy, macronutrient, and micronutrient levels of formulations was unchanged during processing. Reductions in moisture levels were accounted for to determine the levels of raw ingredients required to produce 1 kg and regulated servings of each finished food. Raw ingredients used in traditional and reformulated pan bread were adjusted to 10% moisture in the final product, and the breakfast cereals and pasta were assumed to have lost 70% and 72% moisture during processing, respectively. Accordingly, as per Table 1, the total mass of raw ingredients required to produce 1 kg of each food was ~1.043 kg for pan bread, ~1.123 kg for breakfast cereal, and ~1.257 kg for pasta.

### 2.2. Nutrient Composition of Ingredients

The nutrient composition of raw ingredients is summarized in Supplementary Table S1. Although Canadian regulations stipulate that all refined wheat flour sold and used to manufacture foods is fortified with thiamin, riboflavin, niacin, folic acid, and iron [25,26], Canada is a primary exporter of wheat to other countries for use as ingredients in processed foods, where Canadian-derived wheat flour may or may not be fortified, depending on regional regulations. Thus, the nutritional analysis in this study presents a worst-case scenario regarding the nutritional merits of unfortified refined wheat in processed food products. The nutrient composition for unenriched refined flours were used in this study, and obtained from the US Department of Agriculture (USDA) Food Composition Database (Release 28) [27]. For semolina flour, compositional data corresponding to linoleic acid, trans fat, sugars, vitamin E, vitamin K, choline, and selenium was not provided in the USDA database, and was imputed as the average between industrial high protein and all-purpose flours used for the pan bread and breakfast cereal, respectively (Table S1).

Whole yellow pea flour is obtained from milling dry whole yellow peas (Table S1). The nutrient composition of yellow pea flour was obtained from independent nutritional analysis of whole yellow peas (Silliker Canada Co., Markham, ON, Canada), where it was assumed that negligible nutritional losses occur during the milling process. When the independent analysis did not provide data for specific nutrients, nutrient data for raw split peas from the Canadian Nutrient file (CNF) (CNF #3394) was used [28]. Thus, given that the outer hull accounts for approximately 10% the mass of whole pea, to account the dilution of nutrients by mass, nutrient values derived from the CNF for split peas were decreased by 10%. For the traditional and reformulated foods, it was also assumed that nutrient losses during the manufacturing stage was negligible.

In Canada, the Reference Amount represents a regulated serving of food and was used in this study as the serving sizes for the traditional and reformulated food products. The Reference Amounts are 75 g for pan bread, 30 g for breakfast cereal (low density: 20 g to 42 g per 250 mL; without milk), and 85 g for pasta (dry) [29].

### 2.3. Calculation of the Nutritional Quality of Food

The nutritional quality of traditional and reformulated foods was determined using the nutrient balance concept (NBC) proposed by Fern et al. [30] and nutrient composition data from Table S1. The NBC provides an aggregated measure of nutrients and other dietary constituents considered to have a positive or negative effect on the nutritional profile of a given food. Briefly, the NBC consists of three metrics of nutritional quality: the qualifying index (QI), the disqualifying index (DI), and the nutrient balance score (NBS) [30]. 

The QI is defined as the mean of the ratio of qualifying nutrients contained in 2000 kcal of a given food relative to their daily values (DV) across qualifying nutrients (Equation (1)).

(1) $$QI_{k}=\frac{\frac{2000\;\mathrm{kcal}}{E_{k}}\times \sum_{j=1}^{N_{q}}\frac{a_{q,j}}{DV_{q,j}}}{N_{q}}$$

where *QI_k_* is the *QI* of an individual nutrient food *k*, 2000 kcal represents the total daily energy intake to which nutrition labelling is based in Canada [31], and *E_k_* is the number of calories per serving of food *k* (pan bread: 75g; breakfast cereal: 30 g; pasta (dry): 85 g [29]). The amount of each qualifying nutrient *a* relative to *DV* is represented by *a_q,j_*/*DV_q,j_*. *N_q_* is the number of qualifying nutrients (*q*) considered (*N_q_* = 27). If the *QI* value is >1, the food item is considered nutrient dense; if the *QI* value is <1, the food item is considered to be energy dense [30].

The DVs for qualifying nutrients are summarized in Table 2. DVs are based on Dietary Reference Intakes (DRIs) established by National Academy of Sciences, and are based on the population coverage approach [32]. DV for water, protein, α-linolenic acid, and linoleic acid have not been adopted in Canada [33]. Therefore, for these nutrients, DRIs from the National Academy of Sciences were used and established as the average DVs for men and women ≥19 years of age [34].

The DI represents the levels of 5 public health-sensitive nutrients *d* (sugar, sodium, total fat, saturated fat, and cholesterol) in a food product relative to their daily maximal reference values (MRV). It is calculated as the average of the sum of ratios of disqualifying nutrients to their MRV (Equation (2)) in 2000 kcal food [30].

(2) $$DI_{k}=\frac{\frac{2000\;\mathrm{kcal}}{E_{k}}\times \sum_{j=1}^{N_{d}}\frac{a_{d,j}}{MRV_{d,j}}}{N_{d}}$$

where *DI_k_* is the disqualifying index for food *k*. Again, 2000 kcal represents the total daily energy intake, and *E_k_* is the energy content of a serving of pan bread (75 g), breakfast cereal (30 g), or pasta (85 g). The level of each disqualifying component *a* per MRV within food *k* is represented by *a_d,j_*/*MRV_d,j_*. *N_d_* is the number if disqualifying components in food *k*. MRVs for disqualifying nutrients are summarized in Table 2. Trans fatty acids was not included as a disqualifying nutrient in this study as levels were not available for yellow pea flour in the Canadian Nutrient File, and the Government of Canada has banned the use of partially hydrogenated oils in Canada [35]. Therefore, for this analysis, *N_d_* = 5. If the DI value is > 1, the food item is deemed “compromised” because it contains disqualifying nutrients in values higher than the MRV relative to the energy content of the food item [30].

The third metric, the NBS, was calculated using the average QI of all 27 qualifying nutrients (Equation (3)):

(3) $$NBS_{k}=100\cdot \left(\frac{\sum_{q=1}^{N_{q}}QI_{q,k}}{N_{q}}\right)$$

where *NBS_k_* is the nutrient balance for food *k*. *QI_q,k_* is the qualifying index for each nutrient *q* in food *k. N_q_* = 27 is the number of nutrients identified as qualifying ingredients. As outlined by Fern et al. [30], when calculating the NBS (Equation (3) above), if the *QI_q,k_* for a given nutrient *q* is >1, the QI is truncated to 1, based on the rationale that the requirement for a specific qualifying nutrient is already met. If QI is ≤1, it remains unchanged. An NBS of 100% is achieved if a food satisfies 100% of the daily dietary requirement for every qualifying nutrient in a 2000 kcal diet. Conversely, a value of 0 implies that none of the qualifying nutrients are contained in the diet [30].

### 2.4. Carbon Footprint Analysis of Traditional and Reformulated Foods

It is well known that the agricultural inputs, processes, field operations, and outputs, such as crop yields and emissions, vary widely across the globe, even for a given particular crop. Most of the existing LCA inventory databases provide geographically generic or EU (European Union)-based crop inventory data. Utilizing such data makes the calculated environmental impact a proxy and an estimate. To achieve a more accurate life cycle footprint, regional (spatially-explicit) datasets are required. Therefore, in this study, where possible, crop production, milling, processing, and manufacturing data corresponding to Western Canadian conditions were used. When not available, existing LCA inventory databases provided geographically generic or EU-based crop inventory data.

The carbon footprint of the traditional and reformulated pan bread, pasta, and breakfast cereal was assessed as CO_2_ equivalents (eq). Three supply chain stages for pan bread, pasta, and breakfast cereal were considered for the LCA component of this study: (a) Crop cultivation (yellow peas and wheat), (b) Milling (converting grains into flour) and, (c) Product manufacturing (converting flour and other ingredients into the final product). GHG emissions from packaging, retail, product utilization, and post-consumer recycling were not considered, as these stages are highly site-dependent, and outside the scope of this study. It was assumed that, regardless of the formulation, the emissions from these stages would be largely unchanged across traditional and reformulated foods. Furthermore, within the LCA, these stages often contribute marginally to the total carbon footprint of the foods relative to the production, processing, and manufacturing stages [15].

### 2.5. Greenhouse Gas Emissions from the Cultivation of Yellow Peas and Wheat

Greenhouse gas emissions corresponding to the cultivation of yellow peas and wheat in Western Canada were from a recent systematic review [4]. Briefly, yields of pea (2132 kg/ha) and cereals (2671 kg grain/ha) from a cereal-cereal and oilseed-cereal rotation was from Western Canadian production statistics from 1993 to 2011 [36]. Moreover, cereal data corresponded to wheat and barley. Six sources of production and application-related GHG emissions were compiled: nitrogen fertilizer, phosphorus fertilizer, pesticide, seed production, field operations (e.g., tractor use), and direct/indirect nitrous oxide emissions [4]. Nitrogen and phosphate fertilizer rates were taken from the Canadian Field Print Initiative Fertilizer Use Survey in 2014/2015 [37]. GHG emissions from fertilizer utilization (peas: 0 kg CO_2_ eq/kg N; cereals: 3.48 kg CO_2_ eq/kg N; peas and cereals: 1.62 kg CO_2_ eq/kg P_2_O_5_) were based on emission factors from the DataSmart Life Cycle Inventory [38]. Seed application rates and associated GHG emissions from seed production were based on the Government of Saskatchewan’s Crop Planning Guide [39] and MacWilliam et al. [40], respectively. Emissions from the production, distribution, and use of pesticides (pea 35 kg CO_2_ eq/ha; cereals: 39 kg CO_2_ eq/ha), as well as the production and use of fuel for field operations (planting, tilling, applying pesticides and fertilizers, and harvesting) (peas: 114 kg CO_2_ eq/ha; cereals: 119 kg CO_2_ eq/ha) was derived from studies [41,42,43,44,45,46] included in the systematic review by MacWilliam et al. [4]. Coefficients corresponding to the energy content of diesel fuel (0.0387 GJ/L) [47] and combustion of diesel fuel (CO_2_: 2.7 kg/L; CH_4_: 0.00038 kg/L; N_2_O: 0.000069 kg/L) [48] were used to determine CO_2_ eq associated with field operations. Results demonstrated that GHG emissions associated with the production of dry peas in Western Canada was 0.188 kg CO_2_ eq/kg peas (range: 0.134–0.243 kg CO_2_ eq/kg) [4]. The carbon footprint of cultivating cereals (cereal-cereal and oilseed-cereal rotation) (wheat and barley) was 0.330 kg CO_2_ eq/kg cereals (range: 0.259–0.404 kg CO_2_ eq/kg) and used as a proxy for GHG emissions from wheat grown in Western Canada.

### 2.6. Grain, Energy, and Water Required for Milling Wheat and Yellow Peas

The grain, energy, and water requirements for milling 1 kg of yellow peas and wheat into flour is presented in Supplementary Table S2. Briefly, during the milling of wheat, it was assumed that 3% of wheat grain was rejected for excess moisture, or due to the presence of foreign materials and contamination. Furthermore, a 22% loss of grain occurs during the extraction of the endosperm [49]. Accounting for these losses, 1.32 kg of wheat and 0.134 kW∙h electricity was required to produce 1 kg of all purpose and high-protein bread refined wheat flour [49].

Data from Guidice et al. [50] showed that 1.56 kg of durum semolina, 0.83 kW∙h electricity, and 0.015 kg of water is required to produce 1 kg of durum flour.

For Canadian yellow peas, it was assumed that 1.14 kg whole yellow peas [51] and 0.209 kW∙h electricity [52] was required to produce 1 kg whole yellow pea flour.

### 2.7. Energy and Water Utilization during the Manufacturing of Food Products

A detailed breakdown of the natural gas, electricity, and water required during the manufacturing stage of food production is provided in Table S3. For pan bread, the energy requirements for handling, mixing, dividing, proofing (first and second), molding, baking, and de-panning and cooling were taken into account, based on data provided in Goucher et al. [49].

The energy requirement for the production of extruded breakfast cereal was imported from Jeswani et al. [53], which included cooking in boiled water, cooling, and tempering, as well as rolling, shredding, and cutting. The energy data for pasta production was from Guidice et al. [50]. Other GHG coefficients (kg CO_2_ eq. per kg ingredient) and emissions from the production of non-flour ingredients (e.g., sugar, salt, shortening etc.) were considered during the manufacturing stage of this analysis (Tables S4–S6). For the pan bread, GHG emissions from the production of canola oil was used to estimate the carbon footprint from the use of vegetable shortening.

### 2.8. The Electricity Supply Mixture in Canada

Across the milling and manufacturing stages, data from Itten et al. [54] was used to determine the proportion of renewable and non-renewable sources of electricity per kW∙h in Canada. Table S7 demonstrates that approximately 60% of Canada’s electricity is sourced from renewable resources of energy, particularly hydroelectric power. Carbon emission factors from Ecoinvent (Version 2.2, Swiss Centre for Life Cycle Inventories, Zurich, Switzerland) [55] were used to calculate the CO_2_ eq per kW∙h of each energy resource. The sum, 0.2988 CO_2_ eq/kW∙h (Table S7), was used to convert electricity utilization during the milling and manufacturing stages of pan bread, breakfast cereal, and pasta into GHG emissions.

### 2.9. Determination of the Carbon Footprint of Food Products

The impact assessment method IPCC (2013) for Global Warming Potential (20 years) was used in the present LCA. Carbon emission factors from Ecoinvent (Version 2.2, Swiss Centre for Life Cycle Inventories, Zurich, Switzerland) [55] and the AgriBalyze Database (Version 1.3, SimaPro, Amersfoort, The Netherlands) [56], were used to convert inputs (energy, water, ingredients) from the milling and manufacturing of foods to GHG emissions (CO_2_ eq/unit) (Tables S4–S6).

### 2.10. The Nutrition Carbon Footprint Score

A combined nutrition carbon footprint score (NCFS), as an indicator of product level nutrient density per unit environmental impact, was determined by dividing the NBS by the carbon footprint of the food product (g CO_2_ eq per serving) (Equation (4)).

(4) $$NCFS=\frac{NBS}{\mathrm{Carbon}\;\mathrm{Footprint}\;\left(\frac{g\;{\mathrm{CO}}_{2}\;\mathrm{eq}}{\mathrm{serving}\;\left(g\right)}\right)}$$

## 3. Results

### 3.1. Nutritional Quality Comparison of Traditional and Reformulated Products

Table 3 shows that pan bread, breakfast cereal, and pasta reformulated with whole yellow pea flour have a 13%, 90%, and 30% higher QI, respectively, than corresponding traditional formulations. The increase in QI facilitated an increase in the nutrition balance score (NBS, Equation (3)) across all foods, with the greatest increase observed in breakfast cereals (traditional: 30%; reformulated: 51%; +70%) followed by pasta (traditional: 43%; reformulated: 51%; +18%) and pan bread (traditional: 47%; reformulated: 52%; +11%). In terms of individual nutrients, replacing wheat flour with yellow pea flour increased the amount of 10 out of 27 essential nutrients considered in each product. These were fiber, niacin, pantothenic acid, thiamin, vitamin C, choline, iron, phosphorus, potassium, and zinc. The amount of other 17 nutrients either remained the same, or decreased only marginally, when wheat flour was replaced by yellow pea flour.

Given the absence of disqualifying nutrients in refined wheat flour and whole yellow pea flour, the DI (Equation (2)) was relatively unchanged when foods were reformulated with whole yellow pea flour. Overall, pan bread had the highest NBS, QI, and DI values for both the traditional and reformulated scenarios.

As expected, across all three reformulated food products, as the proportion of the yellow pea flour in the total flour increased from 0 to 100%, the NBS increased linearly (Figure S1). For each 10% wheat flour replaced by whole yellow pea flour, the NBS increased by 1.4% for the pan bread, 2.7% for the breakfast cereal, and 1.6% for the pasta. Generally, when whole yellow pea flour represented between 80% and 90% of the total flour in each formulation, the NBS began to plateau.

### 3.2. Carbon Footprint Comparison of Traditional and Reformulated Food Products

The carbon footprint of products reformulated with yellow pea flour is lower than the traditional products containing wheat flour only (Table 3). The mean carbon footprint of 1 kg of traditional pan bread, breakfast cereal, and pasta was 0.405, 0.979, and 0.610 kg CO_2_ eq/kg, respectively. Overall, reformulation with whole yellow pea flour decreased total GHG emissions by 4% for pan bread, 11% for breakfast cereal, and 13% for pasta. Contrary to emissions data standardized per kg produced, when the carbon footprint per serving size was considered, traditionally formulated pasta represented the food with the largest carbon footprint at 65.20 g CO_2_ eq/85 g (dry) serving (Table 3 and Table S8). Carbon emissions for traditionally formulated pan bread (31.70 g CO_2_ eq/75 g serving) and the breakfast cereal (32.99 g CO_2_ eq/30 g serving) per serving were similar.

The contribution of each life cycle stage to the calculated carbon footprint of foods are presented in Table 4. For traditional pan bread (0.242 kg CO_2_ eq/kg) and pasta (0.518 kg CO_2_ eq/kg), the cultivation of wheat represented 60% and 85% of the total greenhouse gas emissions. However, for traditional breakfast cereal, the high energy requirements during manufacturing stage was the largest contributor to its total carbon footprint at 51% (0.498 kg CO_2_ eq/kg), followed by cultivation of wheat (45%). Given that the carbon footprint for the cultivation of yellow pea (0.188 kg CO_2_ eq/kg) is approximately half the carbon footprint of Canadian wheat from a cereal-cereal and oilseed-cereal rotation (0.33 kg CO_2_ eq/kg) [4], reformulation of pan bread, breakfast cereal, and pasta decreased GHG emissions associated with cultivation by 7%, 26%, and 17%, respectively, and increased the relative contribution of GHG emissions during milling and manufacturing (Table 4 and Table S8).

Figure S2A showed that, for each 10% increase in whole yellow pea flour, GHG emissions per kg food was decreased by 0.011 kg CO_2_ eq for pan bread, 0.020 kg CO_2_ eq for breakfast cereal, and 0.026 kg CO_2_ eq for pasta. Similarly, on a per serving basis, for each 10% increase in whole yellow flour, carbon emissions were decreased by 0.847 g CO_2_ eq (pan bread), 0.665 g CO_2_ eq (breakfast cereal), and 2.784 g CO_2_ eq (pasta) (Figure S2B).

### 3.3. The Combined Nutrition Combined Carbon Footprint Score (NCFS)

When the NBS and carbon footprint data (g CO_2_ eq/serving) were combined, the NCFS (Equation (4)) was highest for the pan bread (traditional: 1.49; reformulated: 1.72) followed by breakfast cereals (traditional: 0.91; reformulated: 1.73), and pasta (traditional: 0.67; reformulated: 0.90) in both traditional (0% pea flour) and reformulated scenarios (Table 3). Given that the traditional and reformulated pan bread had the highest NBS and lowest carbon footprint per serving size, compared to all other foods, it had the highest NCFS. For the breakfast cereal and pasta, there were nutritional and GHG trade-offs. While traditional breakfast cereals have a lower carbon footprint per serving size (32.99 g CO_2_ eq.) than pasta (65.20 g CO_2_ eq.), its NBS (30%) was lower than that of pasta (43%), which moderated the effects of reformulation on the NCFS. Overall, reformulating with yellow pea flour increased the NCFS by 15%, 90%, and 35% for pan bread, breakfast cereals, and pasta, respectively. Figure S3 demonstrates that, each 10% increase in the NCFS increased 0.11 NBS/g CO_2_ eq/serving for pan bread, 0.13 NBS/g CO_2_ eq/serving for breakfast cereal, and 0.09 NBS/g CO_2_ eq/serving for pasta. In addition, when whole yellow pea flour represented 100% of the flour in the formulation, the NCFS was increased to 2.65 (+78%) for the pan bread, 2.19 (+141%) for the breakfast cereal, and 1.58 (+136%) for the pasta, compared to the traditional formulations.

## 4. Discussion

Results from this study demonstrated that replacement of refined wheat flour with whole yellow pea flour can substantially decrease the carbon footprint and concurrently increase the nutritional density of commonly consumed processed vegetarian foods, such as pan bread, breakfast cereal, and pasta. The magnitude of these effects is proportional to the level of pea flour used in product formulations. As introduced by the NCFS, this study also demonstrated that aggregated nutritional profiling systems, such as the NBS, can be combined with LCA analysis to simultaneously evaluate the nutrient density per unit sustainability of food products. These results contribute to the growing body of scientific evidence on the potential for pulses to improve the nutritional [8,9,10] and environmental profile of individual diets and national food systems [4,5,6,7,57,58].

Application of the NBS [30] revealed that the nutritional quality increases almost linearly when the wheat flour is replaced by whole yellow pea flour in reformulated pan bread, breakfast cereal, and pasta. The increase in NBSs across all three products was largely secondary to higher levels of protein, fiber, vitamins, calcium, and potassium in yellow pea flour, than in refined wheat flour.

Globally, food production, and ultimately, consumption, accounts for ~33% of total greenhouse gas emissions [59]. Therefore, knowing where and at what level environmental impacts occur within specific food supply chains is required for farmers, the agri-food industry, and consumers, to collaboratively mitigate the effects of food production on climate change. Diets based on plant-based sources of proteins have been widely documented to have lower carbon footprints than foods containing animal sources of protein [57,58,60].

Breakfast cereals had the largest carbon footprint per kg (Table 3). When GHG emissions were analyzed per serving size, traditional (65.20 g CO_2_ eq/serving) and reformulated pasta (56.85 g CO_2_ eq/serving) displaced breakfast cereal (traditional: 32.99 g CO_2_ eq/serving; reformulated: 29.47 g CO_2_ eq/serving) as the largest producer of GHG emissions (Table S8). This was due to a larger serving size for pasta (85 g dry) compared to breakfast cereal (30 g) and pan bread (75 g). Compared to the breakfast cereal, reformulated pasta also contained a lower proportion of yellow pea flour at 30% total flour. 

With the exception of breakfast cereals, wheat cultivation was the primary contributor of carbon emissions in traditional pan bread, and pasta (Table 4). This was due to the emissions associated with nitrogen fertilizer application, field operations requiring diesel use, phosphate fertilizer, pesticides, and seed application (Tables S4–S6). These large effects of wheat cultivation on the carbon footprint of bread and breakfast cereals is consistent with results from previous LCAs [49,61,62]. Also, given that the GHG emissions from yellow pea production were almost half the carbon footprint of wheat, it was not surprising that GHG emissions from wheat cultivation were decreased when foods were partially reformulated to use whole yellow pea flour. The ability of whole yellow peas to fix nitrogen and lower nitrogen fertilizer requirements per kg produced were the primary factors that lowered cultivation-related GHG emissions in reformulated foods.

Conversely, Table 4 demonstrated that reformulation marginally decreased GHG emissions during the cultivation stage of reformulation in pan bread and pasta. This was largely secondary to the relatively lower inclusion of yellow peas in the reformulated product (15% (pan bread) and 30% (pasta) of total flour). Furthermore, for pasta, 36% of durum wheat grain is lost when milled into durum semolina flour (Table S2: 1.56 kg durum wheat grain per kg durum semolina flour). Processes that recover, and subsequently use these lost fractions of durum grain, were not considered in this LCA, but could credit GHG emissions associated with the production of pasta.

In this study, carbon footprints associated with the cultivation of Canadian wheat was derived from cereal-wheat or oilseed-wheat crop rotation systems. Canadian production data for spring wheat from 2015 shows that 51% of wheat is grown after oilseed, 23% after another cereal, and only 19% was grown after a pulse crop [63]. Compared to a cereal-cereal rotation, MacWilliam et al. [4] demonstrated that GHG emissions from the cultivation stage of cereals is decreased by 65% to 0.166 kg CO_2_ eq/kg if the wheat is preceded by yellow peas or lentils. This is due to the ability of peas and lentils to fix nitrogen, increase the nitrogen levels in the soil, and reduce requirements for synthetic nitrogen fertilizers on subsequent crops. Hence, there is an opportunity to further decrease GHG emissions from wheat by optimizing nitrogen fertility rates when wheat is grown after a nitrogen-fixing pulse crop (Table 3).

Manufacturing accounted for the largest carbon footprint for the production of breakfast cereals, which is due to the high energy requirements required to transform ingredients to the final food product (Table S5). Furthermore, the relative contribution of manufacturing breakfast cereal increased from 51% to 57% when the product was reformulated with pea flour and GHG emissions from cultivation were decreased. The high-energy requirements during the manufacturing stage of breakfast cereal demonstrated that, similar to cultivation, product development is another target for devising strategies to reduce the carbon footprint of food production.

Figures S1–S3 modelled the potential effects of food reformulation on nutrition and environmental outcomes when Canadian whole yellow pea flour ranged from 0% to 100% of total flour. Increasing amounts of whole pea flour in food formulations can present technical challenges during the manufacturing process. However, the baseline recipes developed and used in the present analysis represent a single formulation, which was also constrained by the equipment and manufacturing process available in the facility to which recipes were developed. Given that sustainability benefits of yellow peas are largely realized at the farm-level, although challenging, the data provided in Figures S1–S3 demonstrate conceivable improvements in nutritional density and sustainability of manufactured foods as technology becomes further optimized to permit incremental increases in the use of yellow pea in manufactured foods.

The calculated carbon footprints of the three products in the present study compare well with the previous LCAs. For example, Braschkat et al. [64] demonstrated that greenhouse gas emissions from producing 1 kg of bread through conventional farming and industrial milling produced 0.450 kg CO_2_ eq, which is similar to results from this study (0.405 kg CO_2_ eq/kg). However, Espinoza-Orias et al. [65] showed that the carbon footprint from wheat-based bread was considerably higher than demonstrated in this study, at 0.7–0.9 kg CO_2_ eq/kg. The carbon footprint from LCA for breakfast cereals manufactured in Europe was 1.85 kg CO_2_ eq [53], and is 2× the levels calculated in this study (0.979 kg CO_2_ eq/kg). Nette et al. [66] demonstrated that wheat/egg pasta reformulated to contain (pea flour) decreased GHG emissions 1.22 kg CO_2_ eq/kg, which is, again, much higher than levels in this study. Differences between the present analysis and previous LCA can be attributed to different crop yields, agricultural practices, and electricity mixes between regions.

A lower carbon footprint in Canadian production of wheat is attributed to widespread no-till farm practices that lowers on-farm GHG emissions (e.g., less diesel consumption) [67]. Western Canadian farmers have also adopted cropping system improvements, such as reduced summer fallow, and a general shift away from cereal monoculture rotations, which can further decrease GHG emissions and land degradation [67]. As demonstrated in Table S7, 60% of energy used in Canada is renewable, which is a higher proportion than observed for many other countries [54]. In addition, unlike studies by Espinoza-Orias et al. [65] and Nette et al. [66], emissions associated with packaging, transport, retail, household consumption (e.g., storage energy use) and waste management was not included in the present study, as they were assumed to be neutral when comparing traditional and reformulated food products. It also cannot be overlooked that the LCA by Jeswani et al. [53] included additional ingredients, such as rice, corn, and cocoa, in the formulation of breakfast cereals that also contribute to total carbon footprint of the product.

Rather than site-generic or globally averaged emission factors from different databases [7,52,55], the use of Canada-specific datasets [4] for the analysis of cultivation of wheat and yellow peas is a strength of this carbon footprint analysis. As discussed above, differences in soil, climate, and farming practices affect GHG emissions, and can vary across jurisdictions. A recent analysis by Nortarnicola et al. [68] estimated that the GHG emissions across of 21 types of bread consumed across 21 countries in the EU varied from 0.5 to 6.6 kg CO_2_ eq/kg. The variability was due to the variety of bread and processing requirements across bread types, and suggests the need to use localized datasets as a means to accurately evaluate the product-specific carbon footprints. 

It is increasingly clear that all stakeholders in the global food supply chain, be it on the supply side (producers, traders, and processers) or the demand side (consumers), should act to minimize impacts on the environment [69]. Combining the NBS [30] and life cycle carbon footprint analysis [14,15] to calculate the NCFS for simultaneously evaluating the nutrient density per unit environmental impact per serving is a novel attribute of this study. Moreover, outcomes such as the NCFS, provides a consumer-facing platform to discuss the effect of replacing particular food items on nutritional quality individual carbon footprints. Previous standardization of environmental footprints of food products were based on limited nutrients, such as protein quality [17], which could over- or underestimate the nutritional value of a food per unit carbon. At the same time, depending the nutritional, health, and environmental challenges of a given population or jurisdiction, a more focused approach, such as protein quality, may weight more heavily when evaluating the nutritional implications of food reformulation or replacement. In addition, the identification of carbon emission hotspots (e.g., cultivation and manufacturing stage) in the life cycle of products provides useful information to the producer and processing industries. The quantitative information generated in this study is relevant for stakeholders to collaboratively mitigate the effects of food production on climate change. It is increasingly evident that the food environment is linked to both climate change and chronic disease, and the nutrient density of foods and their carbon footprint should not be studied in isolation.

The present study has limitations and weaknesses that must be taken into account while interpreting the data. First, knowledge gaps exist around the milling and manufacturing stages of food production, depending upon the technology used and which also varies widely between and within countries, and industry stakeholders. Therefore, the published literature was relied upon to provide some of the inputs and carbon emission coefficients (Tables S2–S6) for the three foods evaluated in this study.

Second, it is important to emphasize that unfortified refined wheat flour was used to model the effects of traditional and reformulated foods on nutritional and carbon emissions. Thus, as discussed previously, the present study presents a worst-case scenario for the NBS in traditional and reformulated scenarios. In Canada, it is mandatory that all food products produced with refined wheat flour use wheat flour that is fortified with thiamine, riboflavin, niacin, folic acid, and iron [25,26]. In addition, breakfast cereals and pasta can be voluntarily fortified with thiamin, niacin, folic acid, pantothenic acid, vitamin B_6_, iron, zinc, riboflavin, or magnesium [70]. Therefore, from a nutritional perspective, disparity between traditional and reformulated foods in this study could decrease if fortified Canadian refined wheat flour is considered in the formulation of food products in Canada and other regions.

Third, the NBS does not account for bioavailability of nutrients or protein quality. The latter refers to the ability for dietary protein to be used by humans for metabolic work [71]. Typically, plant-based sources of protein have lower protein quality than animal-derived protein sources, due to challenges around digestibility and lower levels of indispensable amino acids. However, combining cereals and legumes can largely address the issues around protein quality and plant-based foods/diets [72]. Although factors such as nutrient bioavailability and protein quality could affect the absorption and/or assimilation of nutrients, these effects are only meaningful if they are biologically significant.

Finally, the present analysis did not consider the impact of other environmental categories of concern, such as water use [73], land use [74], or biodiversity [75,76,77,78]. Using multiple indicators, a more holistic assessment can better characterize the environmental profile of the food products and also identify trade-offs between impact categories [79]. The scope of this analysis was also confined to daily nutrient requirements outlined by Canadian foods regulations, Canadian cropping systems, and reformulation with one type of pulse (yellow pea). Future studies should explore reformulation analyses for other pulses and food items across different countries, as well as consider the technical and consumer facing (i.e., taste and texture) of producing foods reformulated with high amounts of pea flour.

## 5. Conclusions

Results from this study complement other studies that evaluate the potential of dietary supplements [80], biofortification [81], increased used of pulses [82], nationally recommended diets [83], or nutrient based product labelling schemes [84] on human health and climate change. Broadening the scope of the NCFS framework to evaluate the consequences of food product reformulations could provide valuable insights to global and national policy-makers involved in designing steps towards simultaneous achievement of United Nations’ sustainable development goal (SDG) 3 (good health and well-being), 11 (sustainable cities and communities), 12 (ensuring sustainable consumption and production), and 13 (climate action) [85].

Inclusion of higher amounts of pulses in food products could bring substantial nutritional and environmental advantages, in terms of lower GHG emissions, and a more nutritionally balanced diet. Simultaneous assessment of the effects of food production and reformulation on nutrient density and environmental impact can be used by national and global policy-makers to set quantitative targets (e.g., reducing food related GHG emissions by 20%), evaluate alternative strategies to achieve them (e.g., replacing ruminant meat with dairy products or pulses) while, at the same time, monitor the impact on the nutritional performance of the food systems.

## Figures and Tables

**Table 1:** Mass of raw ingredients (g) required for the production of 1 kg of traditional and reformulated pan bread, breakfast cereal, and pasta.

Ingredient (g)	Pan Bread *		Breakfast Cereal *		Pasta *	
	Traditional	Reformulated	Traditional	Reformulated	Traditional	Reformulated
Whole yellow pea flour	0	83.25	0	536.00	0	301.69
Refined wheat bread flour ^†^	555.00	471.75	0	0	0	0
Refined all-purpose wheat flour, unbleached ^†^	0	0	1011.00	475.33	0	0
Durum Semolina flour ^†^	0	0	0	0	1005.64	703.95
Water	391.36	391.36	53.33	53.33	251.41	251.41
Sugar	22.36	22.36	53.33	53.33	0	0
Shortening ^‡^	22.36	22.36	0	0	0	0
Salt	7.27	7.27	5.33	5.33	0	0
Yeast (fresh)	22.36	22.36	0	0	0	0
Milk powder	11.18	11.18	0	0	0	0
Dough conditioner ^ɣ^	11.18	11.18	0	0	0	0

* Ingredient formulations for reformulated pan bread, breakfast cereal, and pasta were provided by from the Canadian International Grains Institute. All of the flour in traditional formulations was from refined wheat flour. In reformulated foods, a proportion of the total flour was whole yellow pea flour (pan bread: 15%; breakfast cereal: 53%; pasta: 30%). ^†^ Refined wheat bread flour: 12.5–13.5% protein dry weight; refined wheat all-purpose flour: 10.0–12.5% protein dry weight; refined wheat durum flour: 12.0–13.0% protein dry weight [24]. ^‡^ Canola oil was assumed to be used for vegetable shortening. ^ɣ^ Calcium sulfate was assumed to be used as the dough conditioner.

**Table 2:** Summary of daily values (DV) for qualifying nutrients and mean reference values (MRV) for disqualifying nutrients for Canadian adults used to calculate the qualifying index, disqualifying index, and nutrient balance score for reformulated and traditional foods.

Qualifying Nutrient	Daily Value
**Macronutrients**	
Water	3.2 L ^†^
Protein	50 g ^†^
Fiber	28 g *
α-Linolenic Acid	1.4 g ^†^
Linoleic Acid	14 g ^†^
**Vitamins**	
Total folate/folic acid	400 µg *
Niacin	16 mg *
Pantothenic acid	5 mg *
Riboflavin	1.3 mg *
Thiamin	1.2 mg *
Vitamin A	900 µg *
Vitamin B_6_	1.7 mg *
Vitamin B_12_	2.4 µg *
Vitamin C	90 mg *
Vitamin D	20 µg *
Vitamin E	15 mg *
Vitamin K	120 µg *
Choline	550 mg *
**Minerals**	
Calcium	1300 mg *
Copper	0.9 mg *
Iron	18 mg *
Magnesium	420 mg *
Manganese	2.3 mg *
Phosphorous	1250 mg *
Potassium	4700 mg *
Selenium	55 µg *
Zinc	11 mg *
**Disqualifying Nutrients**	**Mean Reference Value per Day**
Sugar	100 g *
Sodium	2300 mg *
Total Fat	75 g *
Saturated Fat	20 g *
Cholesterol	300 mg *

* Government of Canada [33]. ^†^ Daily Reference Intakes (DRIs) established by The National Academy of Sciences were used as daily values for water, protein, α-linolenic acid, and linoleic acid [34].

**Table 3:** The effect of reformulation with whole yellow pea flour on the nutritional profile and carbon footprint of pan bread, breakfast cereals, and pasta.

Product	% of Total Flour per Formulation *		Indices of Nutritional Quality			Carbon Footprint		NCFS
	Wheat Flour	Yellow Pea Flour	QI	DI	NBS (%)	kg CO_2_ eq/kg Food	g CO_2_ eq/Serving ^†^	(NBS/ g CO_2_ eq/Serving)
**Pan Bread**								
Traditional *	100	0	0.62	0.40	47	0.405	31.70	1.49
Reformulated	85	15	0.70	0.40	52	0.389	30.43	1.72
**Breakfast Cereal**								
Traditional	100	0	0.40	0.17	30	0.979	32.99	0.91
Reformulated	47	53	0.76	0.20	51	0.875	29.47	1.73
**Pasta**								
Traditional	100	0	0.57	0.03	43	0.610	65.20	0.67
Reformulated	70	30	0.74	0.04	51	0.532	56.85	0.90

CO_2_ eq: carbon dioxide equivalents; DI: disqualifying index; NBS: nutrient balance score; NCFS: nutrition carbon footprint score; QI: qualifying index. * Traditional formulations contain refined wheat flour (see Table 1). In reformulated foods, a proportion of refined wheat flour was replaced by whole yellow pea flour (15% for pan bread, 53% for the breakfast cereal, and 30% for the pasta). ^†^ Serving sizes correspond to the Reference Amounts outlined by the Government of Canada [29]: pan bread, 75 g; breakfast cereal (low density: 20 g to 42 g per 250 mL; without milk), 30 g; pasta (dry), 85 g.

**Table 4:** Greenhouse gas emissions associated with each stage of the production of 1 kg of traditional and reformulated bread, breakfast cereals, and pasta.

Food	Formulation *	Cultivation Stage		Milling Stage		Manufacturing Stage		Total	
		kg CO_2_ eq	% of Total	kg CO_2_ eq	% of Total	kg CO_2_ eq	% of Total	kg CO_2_ eq	% Difference
**Pan Bread**	Traditional	0.242	60	0.022	5	0.141	35	0.405	
	Reformulated	0.224	58	0.024	6	0.141	36	0.389	−4%
**Breakfast Cereal**	Traditional	0.440	45	0.040	4	0.498	51	0.979	
	Reformulated	0.322	37	0.052	6	0.498	57	0.873	−11%
**Pasta**	Traditional	0.518	85	0.025	4	0.068	11	0.610	
	Reformulated	0.427	80	0.036	7	0.068	13	0.531	−13%

* Traditional formulations contain refined wheat flour (Table 1). In the reformulated foods, a proportion of the total flour was whole yellow pea flour (pan bread: 15%; breakfast cereal, 53%; pasta: 30%). CO_2_ eq, carbon dioxide equivalents.

## Supplementary Materials

The following are available online at http://www.mdpi.com/2072-6643/10/4/490/s1. Table S1: Nutritional composition of raw ingredients (per 100 g) used to produce traditional and reformulated pan bread, breakfast cereals, and pasta. Table S2: Crop and resource inputs required to mill and produce 1kg of refined wheat flour, refined durum semolina flour, and whole yellow pea flour. Table S3: Energy and water required at the manufacturing stage to produce 1 kg of traditional and reformulated pan bread, breakfast cereal, and pasta. Table S4: Summary of inputs and carbon emission factors per unit input used to calculate the GHG emissions from the production of traditional and reformulated pan bread. Table S5: Summary of inputs and carbon emission factors per unit input used to calculate the GHG emissions from the production of traditional and reformulated breakfast cereal. Table S6: Summary of inputs and carbon emission factors per unit input used to calculate the GHG emissions from the production of traditional and reformulated pasta. Table S7: Summary of Canada’s non-renewable and renewable resources used to produce 1 kWh electricity. Table S8: Greenhouse gas emissions associated with each stage of the production per serving of traditional and reformulated bread, breakfast cereals and pasta. Figure S1: Nutrition balance score for pan bread, breakfast cereal, and pasta reformulated with increasing incorporation rates of whole yellow pea flour as a replacement for refined wheat flour. Figure S2: A. Effect of replacing refined wheat flour with whole yellow pea flour on GHG emissions (CO_2_ eq) per kg food produced. B. Effect of replacing refined wheat flour with whole yellow pea flour on GHG emissions (CO_2_ eq) per serving size. Figure S3. Effect of replacing refined wheat flour with whole yellow pea flour on the NCFS.

## References

[1] Economic and Social Development Department, Food and Agriculture Organization of the United Nations (2009). Report of the FAO Expert Meeting on How to Feed the World in 2050.

[2] McDermott J., Wyatt A.J. (2017). The role of pulses in sustainable and healthy food systems. Ann. N. Y. Acad. Sci..

[3] Food and Agriculture Organization of the United Nations Crops Statistics-Concepts, Definitions and Classifications. http://www.fao.org/economic/the-statistics-division-ess/methodology/methodology-systems/crops-statistics-concepts-definitions-and-classifications/en/.

[4] MacWilliam S., Parker D., Tremorin D., Marinangeli C.P.F. White Paper on Carbon Emissions from Pulse Crops. https://www.src.sk.ca/white-paper-carbon-emissions-pulse-crops.

[5] Angadi S.V., McConkey B.G., Cutforth H.W., Miller P.R., Ulrich D., Selles F., Volkmar K.M., Entz M.H., Brandt S.A. (2008). Adaptation of alternative pulse and oilseed crops to the semiarid Canadian Prairie: Seed yield and water use efficiency. Can. J. Plant Sci..

[6] Lupwayi N.Z., Kennedy A.C. (2007). Grain Legumes in Northern Great Plains. Agron. J..

[7] Clune S., Crossin E., Verghese K. (2017). Systematic review of greenhouse gas emissions for different fresh food categories. J. Clean. Prod..

[8] Marinangeli C.P.F., Curran J., Barr S.I., Slavin J., Puri S., Swaminathan S., Tapsell L., Patterson C.A. (2017). Enhancing nutrition with pulses: Defining a recommended serving size for adults. Nutr. Rev..

[9] Mitchell D.C., Lawrence F.R., Hartman T.J., Curran J.M. (2009). Consumption of dry beans, peas, and lentils could improve diet quality in the US population. J. Am. Diet. Assoc..

[10] Mudryj A.N., Yu N., Hartman T.J., Mitchell D.C., Lawrence F.R., Aukema H.M. (2012). Pulse consumption in Canadian adults influences nutrient intakes. Br. J. Nutr..

[11] International Organizaton for Standardization (2006). ISO 14040: Environmental Management—Life Cycle Assessment—Principles and Framework.

[12] International Organizaton for Standardization (2006). ISO 14044: Environmental Management—Life Cycle Assessment—Requirements and Guidelines.

[13] Iasevoli G., Massi M. (2012). The relationship between sustainable business management and competitiveness: Research trends and challenge. Int. J. Technol. Manag..

[14] Chau E.M., Fet A.M. (2008). LCA studies of food products as background for environmental product declarations. Int. J. Life Cycle Assess..

[15] Roy P., Nei D., Orikasa T., Xu Q., Okadome H., Nakamura N., Shiina T. (2009). A review of life cycle assessment (LCA) on some food products. J. Food Eng..

[16] Macdiarmid J.I. (2012). Is a healthy diet an environmentally sustainable diet?. Proc. Nutr. Soc..

[17] Sonesson U., Davis J., Flysjö A., Gustavsson J., Witthöft C. (2017). Protein quality as functional unit—A methodological framework for inclusion in life cycle assessment of food. J. Clean. Prod..

[18] Stylianou K.S., Heller M.C., Fulgoni V.L., Ernstoff A.S., Keoleian G.A., Jolliet O. (2016). A life cycle assessment framework combining nutritional and environmental health impacts of diet: A case study on milk. Int. J. Life Cycle Assess..

[19] Heller M.C., Keoleian G.A., Willett W.C. (2013). Toward a Life Cycle-Based, Diet-level Framework for Food Environmental Impact and Nutritional Quality Assessment: A Critical Review. Environ. Sci. Technol..

[20] Peña-Bautista R.J., Hernandez-Espinosa N., Jones J.M., Guzmán C., Braun H.J. (2017). CIMMYT Series on Carbohydrates, Wheat, Grains, and Health: Wheat-Based Foods: Their Global and Regional Importance in the Food Supply, Nutrition, and Health. Cereal Foods World.

[21] Frohlich P., Borsuk Y., Supeene Y., Sopiwnyk E. (2017). Influence of Pre-Milling Thermal Treatments of Field Peas, Dry Edible Beans and Faba Beans on the Flavour and End Product Quality of Baked Products.

[22] Ebbinghaus P., McMillin K., Hill H., Assefaw E., Sopiwnyk E. (2016). Advancing Pulse Flour Processing and Food Applications Project Report: Pea Based Breakfast Cereal Processing Final Report.

[23] Ebbinghaus P., Bourré L., Maskus H., Assefaw E., Malcolmson L. Influence of Milling Method on the Physical and Functional Properties of Yellow Pea Flour Used in Spaghetti. Proceedings of the AACC International Annual Meeting.

[24] Canadian National Millers Association Generic Product Specifications: Types of Flour According to Use. http://www.canadianmillers.ca/productspecs.php.

[25] Government of Canada Food and Drug Regulations: Division 13, B.13.001-Grain and Bakery Products. http://laws.justice.gc.ca/eng/regulations/c.r.c.,_c._870/page-63.html#h-93.

[26] The Canadian Food Inspection Agency Food Labeling for Industry: Prohibition Against the Sale of Unenriched White Flour and Products Containing Unenriched Flour. http://www.inspection.gc.ca/food/labelling/food-labelling-for-industry/grain-and-bakeryproducts/unenriched-flour/eng/1415915977878/1415915979471.

[27] US Department of Agriculture USDA National Nutrient Database for Standard Reference: Release 28. http://www.ars.usda.gov/Services/docs.htm?docid=8964.

[28] Government of Canada Canadian Nutrient File. https://food-nutrition.canada.ca/cnf-fce/index-eng.jsp.

[29] Government of Canada Table of Reference Amounts for Food. https://www.canada.ca/en/health-canada/services/technical-documents-labelling-requirements/table-reference-amounts-food.html.

[30] Fern E.B., Watzke H., Barclay D.V., Roulin A., Drewnowski A. (2015). The Nutrient Balance Concept: A New Quality Metric for Composite Meals and Diets. PLoS ONE.

[31] The Canadian Food Inspection Agency Food Labeling for Industry: Information within the Nutrition Facts Table—Daily Value and % Daily Value. http://www.inspection.gc.ca/food/labelling/food-labelling-for-industry/nutrition-labelling/information-within-the-nutrition-facts-table/eng/1389198568400/1389198597278?chap=0.

[32] Government of Canada (2016). Regulations Amending the Food and Drug Regulations (Nutrition Labelling, Other Labelling Provisions and Food Colours).

[33] Government of Canada Table of Daily Values. https://www.canada.ca/en/health-canada/services/technical-documents-labelling-requirements/table-daily-values.html.

[34] National Academy of Sciences Table: DRI Values Summary. http://www.nationalacademies.org/hmd/~/media/Files/Activity%20Files/Nutrition/DRI-Tables/5Summary%20TableTables%2014.pdf?la=en.

[35] Government of Canada Notice of Modification—Prohibiting the Use of Partially Hydrogenated Oils (PHOs) in Foods. https://www.canada.ca/en/health-canada/services/food-nutrition/public-involvement-partnerships/modification-prohibiting-use-partially-hydrogenated-oils-in-foods/information-document.html.

[36] Statistics Canada Estimated Areas, Yield, Production and Average Farm Price of Principal Field Crops, in Metric Units. http://www5.statcan.gc.ca/cansim/a26?lang=eng&id=10017.

[37] Canadian Field Print Initiative Fertilizer Use Survey 2014–2015. http://fieldprint.ca/fertilizer-use-survey/.

[38] Long Trail Sustainability DataSmart LCI Package (US-EI SimaPro^®^ Library). https://ltsexperts.com/services/software/datasmart-life-cycle-inventory/.

[39] Government of Saskatchewan Crop Planning Guide Saskatchewan Ministry of Agriculture. http://publications.gov.sk.ca/documents/20/87217-Crop%20Planning%20Guide%202016%20web%20final.pdf.

[40] MacWilliam S., Wismer M., Kulshreshtha S. (2014). Life cycle and economic assessment of Western Canadian pulse systems: The inclusion of pulses in crop rotations. Agric. Syst..

[41] Bremer E., Janzen H.H., Ellert B.H., McKenzie R.H. (2011). Carbon, Nitrogen, and Greenhouse Gas Balances in an 18-Year Cropping System Study on the Northern Great Plains. Soil Sci. Soc. Am. J..

[42] Gan Y., Liang C., Hamel C., Cutforth H., Wang H. (2011). Strategies for reducing the carbon footprint of field crops for semiarid areas. A review. Agron. Sustain. Dev..

[43] Gan Y., Liang C., Wang X., McConkey B. (2011). Lowering carbon footprint of durum wheat by diversifying cropping systems. Field Crop. Res..

[44] Sainju U.M., Barsotti J.L., Wang J. (2014). Net Global Warming Potential and Greenhouse Gas Intensity Affected by Cropping Sequence and Nitrogen Fertilization. Soil Sci. Soc. Am. J..

[45] Zentner R.P., Campbell C.A., Biederbeck V.O., Miller P.R., Selles F., Fernandez M.R. (2001). In Search of a Sustainable Cropping System for the Semiarid Canadian Prairies. J. Sustain. Agric..

[46] Zentner R.P., Lafond G.P., Derksen D.A., Nagy C.N., Wall D.D., May W.E. (2004). Effects of tillage method and crop rotation on non-renewable energy use efficiency for a thin Black Chernozem in the Canadian Prairies. Soil Tillage Res..

[47] Government of Canada Energy Conversion Tables. https://www.neb-one.gc.ca/nrg/tl/cnvrsntbl/cnvrsntbl-eng.html.

[48] United States Environmental Protection Agency (2016). Emission Factors for Greenhouse Gas Inventories.

[49] Goucher L., Bruce R., Cameron D.D., Lenny Koh S.C., Horton P. (2017). The environmental impact of fertilizer embodied in a wheat-to-bread supply chain. Nat. Plants.

[50] Guidice A.L., Clasadonte M.T., Matarazzo A. (2011). LCI Preliminary Results in the Sicilian Durum Wheat Pasta Chain Production. J. Commod. Sci. Technol. Qual..

[51] Maskus H. (2014). Pulse Milling Project Year 4: Summary of Results.

[52] Blonk Consultants Agri-Footprint 2. Part 2: Description of Data. https://simapro.com/wp-content/uploads/2016/03/Agri-footprint-2.0-Part-2-Description-of-data.pdf.

[53] Jeswani H.K., Burkinshaw R., Azapagic A. (2015). Environmental sustainability issues in the food-energy-water nexus: Breakfast cereals and snacks. Sustain. Prod. Consum..

[54] Itten R., Frischknecht R., Stucki M. (2012). Life Cycle Inventories of Electricity Mixes and Grid.

[55] Althaus H.-J., Bauer C., Doka G., Dones R., Frischknecht R., Hellweg S., Humbert S., Jungbluth N., Köllner T. (2010). Implementation of Life Cycle Impact Assessment Methods.

[56] Koch P., Salou T. (2016). Agribalyse^®^: Rapport Methodology.

[57] Foyer C.H., Lam H.M., Nguyen H.T., Siddique K.H., Varshney R.K., Colmer T.D., Cowling W., Bramley H., Mori T.A., Hodgson J.M. (2016). Neglecting legumes has compromised human health and sustainable food production. Nat. Plants.

[58] Sabate J., Soret S. (2014). Sustainability of plant-based diets: Back to the future. Am. J. Clin. Nutr..

[59] Tubiello F.N., Salvatore M., Ferrara A.F., House J., Federici S., Rossi S., Biancalani R., Condor Golec R.D., Jacobs H., Flammini A. (2015). The Contribution of Agriculture, Forestry and other Land Use activities to Global Warming, 1990–2012. Glob. Chang. Biol..

[60] Heller M.C., Keoleian G.A. (2015). Greenhouse Gas Emission Estimates of U.S. Dietary Choices and Food Loss. J. Ind. Ecol..

[61] Kulak M., Nemecek T., Frossard E., Chable V., Gaillard G. (2015). Life cycle assessment of bread from several alternative food networks in Europe. J. Clean. Prod..

[62] Jensen J.K., Arlbjørn J.S. (2014). Product carbon footprint of rye bread. J. Clean. Prod..

[63] Agriculture Agri-Food Canada (2018). Data Requested from Agriculture Agri-Food Canada: Crops That Preceded Spring Wheat Grown in Canada.

[64] Braschkat J., Patyk A., Quirin M., Reinhardt G.A. Life cycle assessment of bread production—A comparison of eight different scenarios. Proceedings of the 4th International Conference on: Life Cycle Assessment in the Agri-food sector.

[65] Espinoza-Orias N., Stichnothe H., Azapagic A. (2011). The carbon footprint of bread. Int. J. Life Cycle Assess..

[66] Nette A., Wolf P., Schluter O., Meyer-Aurich A. (2016). A Comparison of Carbon Footprint and Production Cost of Different Pasta Products Based on Whole Egg and Pea Flour. Foods.

[67] Awada L., Lindwall C.W., Sonntag B. (2014). The development and adoption of conservation tillage systems on the Canadian Prairies. Int. Soil Water Conserv. Res..

[68] Notarnicola B., Tassielli G., Renzulli P.A., Monforti F. (2017). Energy flows and greenhouses gases of EU (European Union) national breads using an LCA (Life Cycle Assessment) approach. J. Clean. Prod..

[69] Chaudhary A., Pfister S., Hellweg S. (2016). Spatially Explicit Analysis of Biodiversity Loss Due to Global Agriculture, Pasture and Forest Land Use from a Producer and Consumer Perspective. Environ. Sci. Technol..

[70] The Canadian Food Inspection Agency Foods to Which Vitamins, Mineral Nutrients and Amino Acids May or Must Be Added [D.03.002, FDR]. http://www.inspection.gc.ca/food/labelling/food-labelling-for-industry/nutrient-content/reference-information/eng/1389908857542/1389908896254?chap=1.

[71] Millward D.J., Layman D.K., Tome D., Schaafsma G. (2008). Protein quality assessment: Impact of expanding understanding of protein and amino acid needs for optimal health. Am. J. Clin. Nutr..

[72] Marsh K.A., Munn E.A., Baines S.K. (2013). Protein and vegetarian diets. Med. J. Aust..

[73] Vanham D., Hoekstra A.Y., Bidoglio G. (2013). Potential water saving through changes in European diets. Environ. Int..

[74] Alexander P., Brown C., Arneth A., Finnigan J., Rounsevell M.D.A. (2016). Human appropriation of land for food: The role of diet. Glob. Environ. Chang..

[75] Chaudhary A., Kastner T. (2016). Land use biodiversity impacts embodied in international food trade. Glob. Environ. Chang..

[76] Chaudhary A., Verones F., de Baan L., Hellweg S. (2015). Quantifying Land Use Impacts on Biodiversity: Combining Species–Area Models and Vulnerability Indicators. Environ. Sci. Technol..

[77] Chaudhary A., Brooks T.M. (2017). National Consumption and Global Trade Impacts on Biodiversity. World Dev..

[78] Chaudhary A., Pourfaraj V., Mooers A.O., Cowie R. (2018). Projecting global land use-driven evolutionary history loss. Divers. Distrib..

[79] Chaudhary A., Gustafson D., Mathys A. (2018). Multi-indicator sustainability assessment of global food systems. Nat. Commun..

[80] Blumberg J.B., Frei B., Fulgoni V.L., Weaver C.M., Zeisel S.H. (2017). Contribution of Dietary Supplements to Nutritional Adequacy in Race/Ethnic Population Subgroups in the United States. Nutrients.

[81] Haas J.D., Luna S.V., Lung’aho M.G., Wenger M.J., Murray-Kolb L.E., Beebe S., Gahutu J.B., Egli I.M. (2016). Consuming Iron Biofortified Beans Increases Iron Status in Rwandan Women after 128 Days in a Randomized Controlled Feeding Trial. J. Nutr..

[82] Abdullah M.M.H., Marinangeli C.P.F., Jones P.J.H., Carlberg J.G. (2017). Canadian Potential Healthcare and Societal Cost Savings from Consumption of Pulses: A Cost-Of-Illness Analysis. Nutrients.

[83] Hendrie G.A., Ridoutt B.G., Wiedmann T.O., Noakes M. (2014). Greenhouse gas emissions and the Australian diet—Comparing dietary recommendations with average intakes. Nutrients.

[84] Lawrence M.A., Dickie S., Woods J.L. (2018). Do Nutrient-Based Front-of-Pack Labelling Schemes Support or Undermine Food-Based Dietary Guideline Recommendations? Lessons from the Australian Health Star Rating System. Nutrients.

[85] United Nations (2015). Transforming Our World: The 2030 Agenda for Sustainable Development.

