# Supplementary material for: Nutritional Combined Greenhouse Gas Life Cycle Analysis for Incorporating Canadian Yellow Pea into Cereal-Based Food Products
Source: Nutrients. 2018 Apr 16;10(4):490. doi: 10.3390/nu10040490 (PMC5946275; doi:10.3390/nu10040490)
Supplement: Supplementary file 1 [file nutrients-10-00490-s001.pdf]

Nutritional combined Greenhouse Gas Life Cycle Analysis for Incorporating Canadian Yellow Pea Flour into Cereal-Based Food Products

Abhishek Chaudhary[1*], Christopher P.F. Marinangeli[2], Denis Tremorin[2] and Alexander Mathys[1]

[1]Institute of Food, Nutrition and Health, ETH Zurich; Switzerland 8092;
[2]Pulse Canada, Winnipeg MB, R3M 0A5, Canada;
*Correspondence: abhishek.chaudhary@hest.ethz.ch; Tel.: +41-44-632-7628

Supplementary Material

Table S1. Nutritional composition of raw ingredients (per 100g) used to produce traditional and reformulated pan bread, breakfast cereals, and pasta.

**Nutrient content per 100 g**	**Unenriched White Wheat All Purpose Flour**	**Unenriched White Wheat Bread Flour (13% protein)**	**Unenriched Semolina Flour**	**Whole Yellow pea Flour**	**White Sugar**	**Vegetable Shortening**	**Yeast**	**Skim Milk Powder**	**Salt**
**Source:**	USDA #: 20481[*]	USDA #: 20641[*]	USDA #: 20466[*]	Independent Analysis[†]	CNF#: 4318[‡]	CNF#: 552[‡]	CNF#: 4008[‡]	CNF#: 134[‡]	CNF#: 214[‡]
Energy (kcal)	364	362	360	354	387	884	325	363	0
Water (g)	11.92	12.82	12.67	10.36	0.02	0	5.08	3.16	0
Protein (g)	10.33	13.07	12.68	18.66	0.00	1.00	40.44	36.16	0
Fibre (g)	2.7	2.4	3.9	23.4	0.0	0.0	26.9	0.0	0.0
Total Fat (g)	0.98	1.38	1.05	1.58	0	100 g	7.61	0.77	0
α-Linolenic Acid (mg)	0.035[¶]	0.042[¶]	0.030[¶]	0.076[ɣ]	0	1.625	1	0.01	0
Linoleic Acid (mg)	0.705[¶]	0.802[¶]	0.7535[¥]	NP[ɣ§]	0	0	NP[§]	NP[§]	0
Trans Fat (g)	0.003[¶]	0.003[¶]	0.003[¥]	NP[ɣ§]	0	16.921	NP[§]	NP[§]	0
Saturated Fat (g)	0.155	0.189	0.15	0.35	0	26.099	1.001	0.499	0
Cholesterol (mg)	0.0	0.0	0.0	0	0	0	0	20	0
Sugar (g)	0.27	1.1	0.685[¥]	2.2	96.75	0	0	51.98	0

**Vitamins**									
Total Folacin (µg)	26	31	72	43.4	0	0	2340	50	0
Niacin (mg)	1.25	1.198	3.31	6.79Ɣ	0	0	40.2	0.951	0
Pantothenic acid (mg)	0.438	0.386	0.58	1.60Ɣ	0	0	13.5	3.568	0
Riboflavin (mg)	0.04	0.072	0.08	0.20Ɣ	0	0	4	1.55	0
Thiamin (mg)	0.12	0.194	0.28	0.66Ɣ	0	0	10.99	0.415	0
Vitamin A as RAE (µg)	0.0	0.0	0.0	0	0	0	0	677	0
Vitamin B_6 (mg)	0.044	0.045	0.103	0.158Ɣ	0	0	1.5	0.361	0
Vitamin B_{12} (µg)	0.0	0.0	0.0	0	0	0	0.07	4.03	0
Vitamin C (mg)	0.0	0.0	0.0	4.9	0	0	0.3	6.8	0
Vitamin D (µg)	0.0	0.0	0.0	0.0Ɣ	0	0	0	10.7	0
Vitamin E (mg)	0.06	0.05	0.055¥	0.08Ɣ	0	0	0	1	0
Vitamin K (µg)	0.3	0.3	0.3¥	13.2Ɣ	0	0	0.4	0.1	0
Choline (mg)	10.4	10.4	10.4¥	86.8Ɣ	NP§	0	NP§	169.2	0
**Minerals**									
Calcium (mg)	15	24	17	63	1	0	30	1257	24
Copper (mg)	0.144	0.187	0.189	0.741Ɣ	0.007	0	0.436	0.041	0.03
Iron (mg)	1.17	1.26	1.23	3.8	0.05	0	2.17	0.32	0.33
Magnesium (mg)	22	35	47	23.4	0	0	54	110	1
Manganese (mg)	0.682	0.624	0.619	1.11Ɣ	0	0	0.312	0.02	0.10
Phosphorous (mg)	108	119	136	283	0	0	637	968	0
Potassium (mg)	107	128	186	961	2	0	955	1794	8
Selenium (µg)	33.9	26.2	30.1¥	3.7Ɣ	0.6	0	7.9	27.3	0.1
Sodium (mg)	2	2	1	8.4	1	0	51	535	38758
Zinc (mg)	0.7	1.6	1.05	2.36	0.01	0	7.94	4.08	0.1

Abbreviations: CNF, Canadian Nutrient File; NP, not provided; RAE, retinol activity equivalents; USDA, US Department of Agriculture

*Unless otherwise indicated, nutrient composition data was provided by the USDA National Nutrient Database for standard reference [1]

† Unless otherwise indicated, nutrient composition data was provided by independent nutrient analysis (Silliker Canada Co., Markham, Ontario Canada) for whole yellow peas was used as proxy for whole yellow pea flour where it was assumed that nutrient losses during milling was negligible.

‡Unless otherwise indicated, nutrient composition data was provided by the CNF [2]

[¶]Levels of these nutrients were not provided in the USDA Food Composition Database (Release 28). Missing values for unenriched all-purpose flour was imputed from the CNF (CNF#6642: Grains, wheat flour, white, all purpose, unbleached). Missing values for unenriched industrial wheat flour (13% protein) was imputed from the CNF (CNF#4445: Grains, wheat flour, white, bread flour). Missing values for unenriched semolina flour was imputed from the CNF (CNF#4478: Grains, semolina).

[¥]Levels of these nutrients within unenriched semolina flour was not reported in the USDA Food Composition Database (Release 28) [1]. Missing values for these nutrients was imputed as the average between unenriched all-purpose flour (USDA# 20481) and unenriched industrial wheat flour (13% protein) (USDA# 20641) [1].

[ɣ]Data corresponding to these nutrients were not provided by the independent analysis of whole yellow peas and was imputed from data corresponding to raw split peas from the CNF (File #3394) [2]. Given that split peas have had the outer hull removed, nutrient values derived from the CNF were reduced by 10% which represents the % mass of the hull on a whole dried yellow pea.

[§]When data for nutrients was not provided, as denoted by "NP," the value was assumed to be 0.0.

Table S2. Crop and resource inputs required to mill and produce 1kg of refined wheat flour, refined durum semolina flour, and whole yellow pea flour.

Production Inventory	Unit	Refined Wheat flour*	Refined Durum semolina	Whole Yellow Pea flour
Wheat Grain	kg	1.32	0.0	0.0
Durum Grain	kg	0.0	1.56	0.0
Dry pea	kg	0.0	0.0	1.14†
Electricity	kwh	0.134	0.083	0.209‡
Water	g	0.0	15.0	0.0
Data sources		Goucher et al. [3]	Guidice et al. [4]	†Maskus [5]; ‡Blonk Agri-footprint, [6]

* Refined wheat flour refers to the high protein bread flour (12.5% - 15.5%) used to produce the pan bread and the refined all-purpose wheat flour (1.0-12.5% protein) used to produce the breakfast cereal (Table S1).

Table S3. Energy and water required at the manufacturing stage to produce 1 kg of traditional and reformulated pan bread, breakfast cereal, and pasta.

Ingredient	Pan bread†		Breakfast cereal†		Pasta†	
	Traditional	Reformulated	Traditional	Reformulated	Traditional	Reformulated
Natural gas (MJ)*	0.0456	0.0456	4.57	4.57	0.00	0.00
Electricity (kWh)*	0.10	0.10	0.42	0.42	0.23	0.23
Water (g) ‡	391.36	391.36	53.33	53.33	251.41	251.41

Abbreviations: kWh, kilowatt hour; MJ, megajoule;

*Electricity and natural gas requirements per kg of bread, breakfast cereals and pasta were from Goucher et al. [3], Jeswani et al. [7] and Guidice et al. [4], respectively.

†All of the flour in traditional formulations was from refined wheat flour (Table 1). In reformulated foods, a proportion of the total flour was whole yellow pea flour (Pan bread: 15%; Breakfast cereal: 53%; Pasta: 30%).

‡ Table 1

Table S4. Summary of input amounts and carbon emission factors per unit input used to calculate the GHG emissions from the production of traditional and reformulated pan bread.

Production Stage	Unit	Level	Emission factor (kgCO2eq/unit)	GHG Emissions (kgCO2eq)
**Cultivation (per kg flour)**				
Wheat grain per kg refined wheat flour	kg	1.3200[*]	0.3300[ˠ]	0.4356
Whole yellow per kg whole yellow pea flour	kg	1.1400[*]	0.1880[ˠ]	0.2143
**Milling (per kg flour)**				
Electricity required per kg wheat all-purpose flour	kWh	0.1340[*]	0.2988[**]	0.0400
Electricity required per kg whole yellow pea flour	kWh	0.2090[*]	0.2988[**]	0.0624
**Manufacturing (per Kg bread)**				
Natural gas (burned in furnace >100kW low nox)	MJ	0.0456[†]	0.0778[¥]	0.0035
Electricity, (Canadian Supply mix)	kWh	0.1000[†]	0.2988[**]	0.0299
Tap Water	kg	0.3914[†‡]	0.0004[¥]	0.0001
Shortening (Canola oil, crude, conventional, animal feed, at plant)	kg	0.0224[‡]	0.8946[§]	0.0200
Salt (Sodium chloride, powder, at plant, processing)	kg	0.0073[‡]	0.1930[¥]	0.0014
Yeast paste	kg	0.0224[‡]	1.1574[¥]	0.0259
Sugar (sugarcane, animal feed, at sugar plant)	kg	0.0224[‡]	0.3085[¥]	0.0069
Skimmed milk powder (feed plant)	kg	0.0112[‡]	4.7780[§]	0.0534
Dough conditioner (Calcium sulfate)	kg	0.0112[‡]	0.0021[¥]	0.000024
**Total Emissions from Manufacturing**				**0.141**

Abbreviations: kWh, kilowatt hour; MJ, megajoule

Sources: [*]Table S2; [†]Table S3; [‡]Table 1; [ˠ]MacWilliam et al.[8]; [**]Table S7; [¥] Hegger and Hischier[9]; [§]Koch and Salou [10].

Table S5. Summary of input amounts and carbon emission factors per unit input used to calculate the GHG emissions from the production of traditional and reformulated breakfast cereal.

Production Stage	Unit	Amount	Emission factor (kgCO2eq/unit)	GHG Emissions (kgCO2eq)
**Cultivation (per kg flour)**				
Wheat grain per kg refined wheat flour	kg	1.3200[*]	0.3300 [Ɣ]	0.4356
Whole yellow per kg whole yellow pea flour	kg	1.1400[*]	0.1880 [Ɣ]	0.2143
**Milling (per kg flour)**				
Electricity required per kg wheat all-purpose flour	kWh	0.1340[*]	0.2988[**]	0.0400
Electricity required per kg whole yellow pea flour	kWh	0.2090[*]	0.2988[**]	0.0624
**Manufacturing (per Kg breakfast cereal)**				
Natural gas (burned in furnace >100kW low nox)	MJ	4.5720[†]	0.0778[¥]	0.3555
Electricity (Canadian supply mix)	kWh	0.4200[†]	0.2988[**]	0.1255
Tap water	kg	0.0533[†‡]	0.0004[¥]	0.000019
Salt (sodium chloride, powder, at plant, processing)	kg	0.0053[‡]	0.1930[¥]	0.0010
Sugar (from sugarcane, animal feed, at sugar plant)	kg	0.0533[‡]	0.3085[¥]	0.0164
**Total Emissions from Manufacturing**				**0.498**

Abbreviations: kWh, kilowatt hour; MJ, megajoule

Source: [*]Table S2; [†]Table S3; [‡]Table 1; [Ɣ]MacWilliam et al.[8]; [**]Table S7; [¥] Hegger and Hischier [9].

Table S6. Summary of input amounts and carbon emission factors per unit input used to model the GHG emissions from the production of traditional and reformulated pasta.

Production Stage	Unit	Amount	Emission factor (kgCO2eq/unit)	GHG Emissions (kgCO2eq)
**Cultivation (per kg flour)**				
Durum wheat grain per kg refined semolina flour	kg	1.5600[*]	0.330[Ɣ]	0.5148
Whole yellow per kg whole yellow pea flour	kg	1.1400[*]	0.188[Ɣ]	0.2143
**Milling (per kg flour)**				
Electricity required per kg semolina flour	kWh	0.0830[*]	0.2988[**]	0.0248
Electricity required per kg whole yellow pea flour	kWh	0.2090[*]	0.2988[**]	0.0624
Water required per kg semolina flour	kg	0.0150[*]	0.0004[¥]	0.0000
**Manufacturing (per Kg pasta)**				
Electricity (Canadian supply mix)	kWh	0.2260[†]	0.2988[**]	0.0675
Tap Water	kg	0.2514[†‡]	0.0004[¥]	0.000088
**Total Emissions from Manufacturing**				**0.068**

Abbreviations: kWh, kilowatt hour; MJ, megajoule

Source: [*]Table S2; [†]Table S3; [‡]Table 1; [Ɣ]MacWilliam et al.[8]; [**]Table S7; [¥]Hegger and Hischier [9]

Table S7. Summary of Canada's non-renewable and renewable resources used to produce 1kWh electricity.

Electricity Source	kWh/Total kWh Supplied*	Emission factor (kgCO2eq/kWh)†	CO2eq/Total kWh
**Non-Renewable Energy**			
Hard Coal	1.348E-01	1.260E+00	1.698E-01
Lignite	2.320E-02	1.248E+00	2.895E-02
Coke	1.000E-04	1.822E+00	1.822E-04
Oil	1.220E-02	9.078E-01	1.108E-02
Natural Gas	5.730E-02	8.448E-01	4.841E-02
Nuclear	1.350E-01	1.411E-02	1.904E-03
Diesel	1.700E-03	4.369E-01	7.427E-04
**Total (%)**	**3.643E-01 (36%)**		
**Renewable Energy**			
Hydro			
Non Alpine	1.158E-01	2.630E-02	3.046E-03
Runoff	4.634E-01	3.990E-03	1.849E-03
Pumped storage	1.681E-04	1.191E+00	2.001E-04
Wind	5.900E-03	1.276E-02	7.531E-05
Photovoltaic	4.745E-05	7.144E-02	3.390E-06
Biomass	1.170E-02		
Wood	1.062E-02	3.192E-02	3.390E-04
Biogas	1.087E-03	5.429E-01	5.899E-04
Incineration	2.000E-04	5.072E-01	1.014E-04
**Total (%)**	**6.087E-01 (60%)**		
**Other**			
Imported (%)	3.850E-02 (4%)	8.192E-01	3.154E-02
**Total**	**1.012**		**0.2988**

Abbreviations: CO_2eq, carbon dioxide equivalents

*Itten et al. [11]

†Hegger and Hischier [9]

Table S8. Greenhouse gas emissions associated with each stage of the production per serving of traditional and reformulated bread, breakfast cereals and pasta.

**Food**	**Formulation***	**Cultivation stage**		**Milling stage**		**Manufacturing stage**		**Total**	
		**gCO2eq/ serving**	**% of Total**	**gCO2eq/ serving**	**% of Total**	**gCO2eq/ serving**	**% of Total**	**gCO2eq/ serving**	**% Difference**
**Pan bread†**	Traditional	18.91	60%	1.74	5%	11.05	35%	31.70	
	Reformulated	17.52	58%	1.88	6%	11.05	36%	30.45	-4%
**Breakfast Cereal†**	Traditional	14.84	45%	1.36	4%	16.79	51%	32.99	
	Reformulated	10.85	37%	1.77	6%	16.79	57%	29.41	-11%
**Pasta†**	Traditional	55.32	85%	2.66	4%	7.22	11%	65.20	
	Reformulated	45.63	80%	3.89	7%	7.22	13%	56.73	-13%

*All of the flour in traditional formulations was from refined wheat flour (Table 1). In reformulated foods, a proportion of total flour that was whole yellow pea flour (Pan bread: 15%; Breakfast cereal: 53%; Pasta: 30%).

†Serving sizes corresponded to Reference Amounts provided by the Government of Canada (Pan bread, 75 g; Breakfast cereal (low density: 20 g to 42 g per 250 mL; without milk): 30 g; Pasta (dry): 85 g) [12].

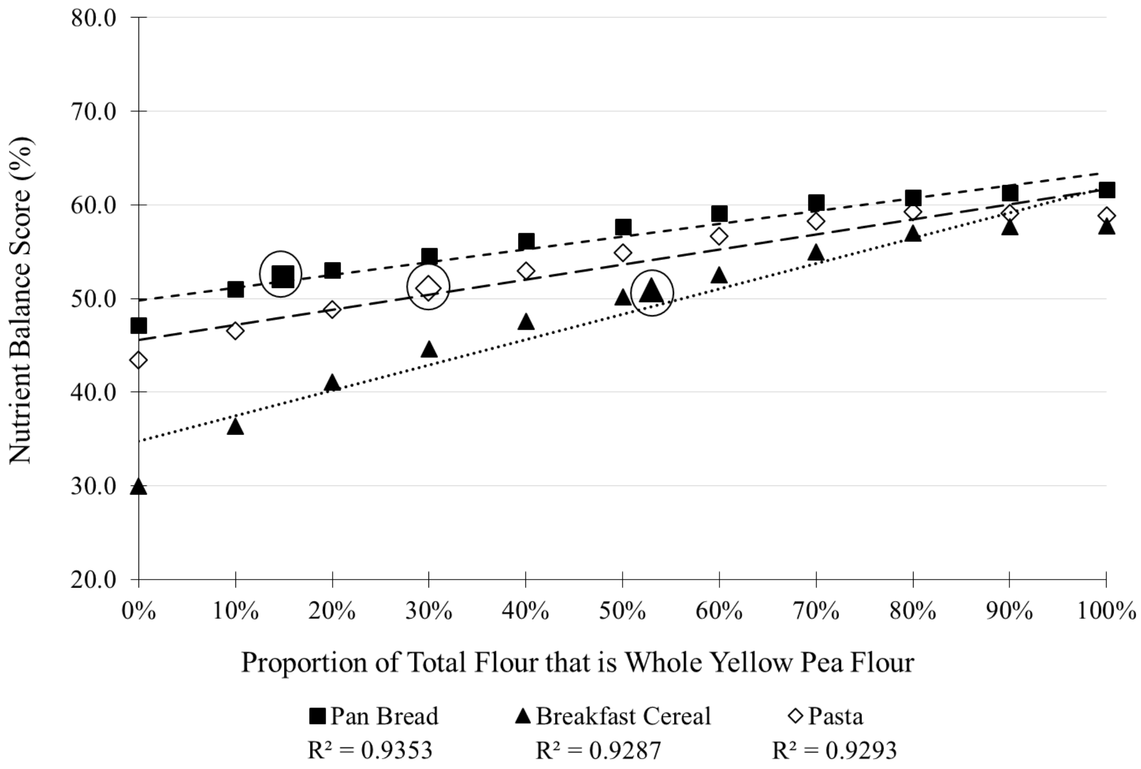

Figure S1. Nutrition Balance Score for pan bread, breakfast cereal, and pasta reformulated with increasing incorporation rates of whole yellow pea flour as a replacement for refined wheat flour. Enlarged markers that are circled represent the baseline reformulated foods where the proportion of the total flour was yellow pea flour (Pan bread: 15%; Breakfast cereal: 53%; Pasta: 30%).

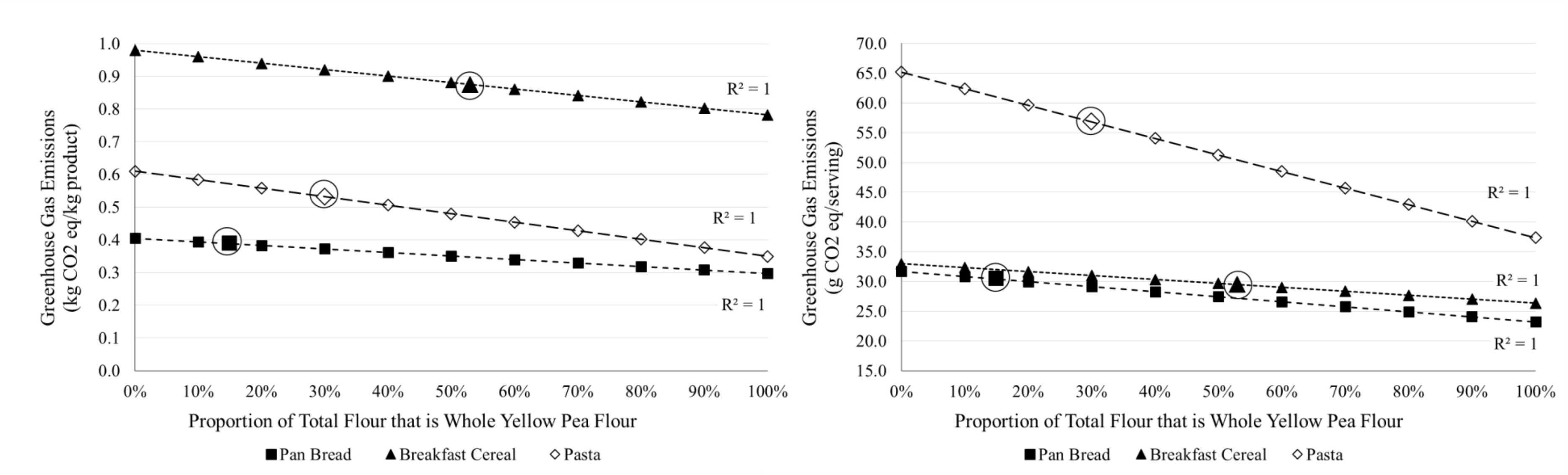

Figure S2. A. Effect of replacing refined wheat flour with whole yellow pea flour on GHG emissions (CO_2eq) per kg food produced. **B.** Effect of replacing refined wheat flour with whole yellow pea flour on GHG emissions (CO_2eq) per serving size. Enlarged markers that are circled represent the baseline reformulated foods where a proportion total flour in the formulations was whole yellow pea flour (Pan bread: 15%; Breakfast cereal, 53%; Pasta: 30%).

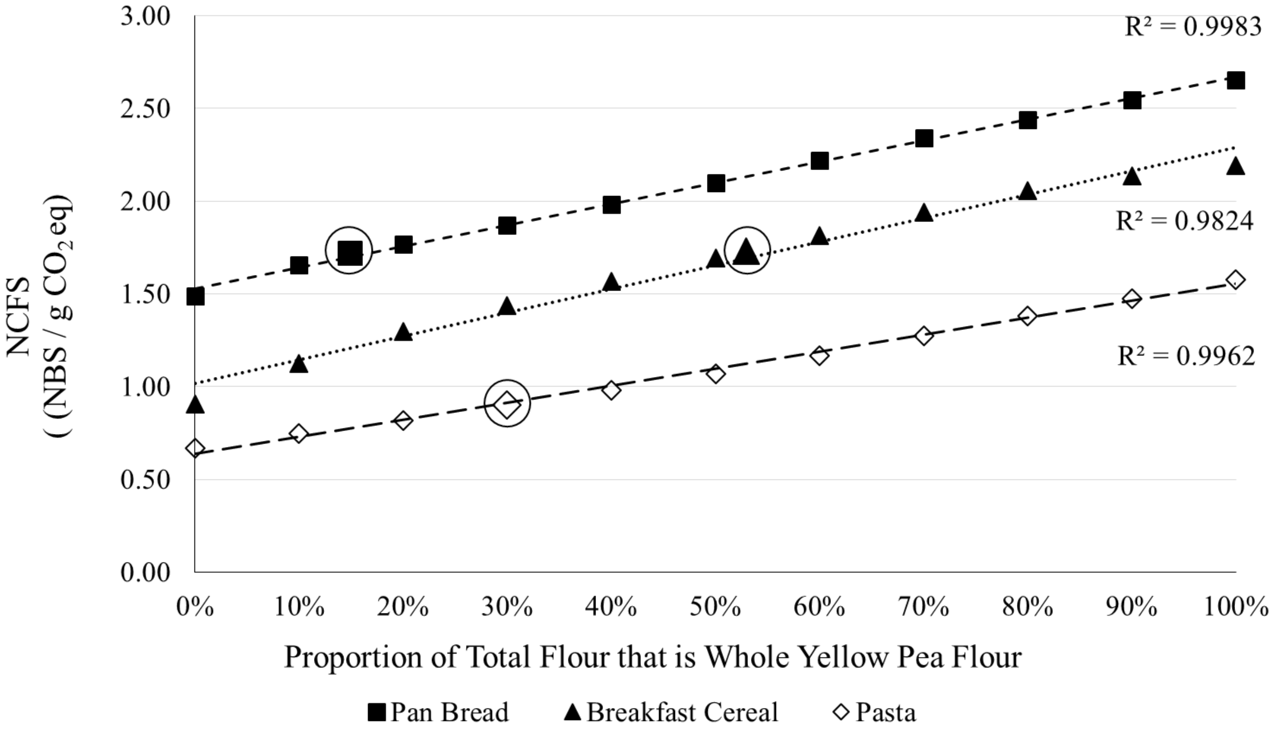

Figure S3. Effect of replacing refined wheat flour with whole yellow pea flour on the NCFS. Enlarged markers that are circled represent the baseline reformulated foods where a proportion total flour in the formulations was whole yellow pea flour (Pan bread: 15%; Breakfast cereal, 53%; Pasta: 30%). NCFS, nutrition carbon footprint score (NBS/ gCO2eq/serving).

References

1. US Department of Agriculture. USDA National Nutrient Database for standard reference: Release 28. http://www.ars.usda.gov/Services/docs.htm?docid=8964 (March, 2017).

2. Government of Canada. Canadian Nutrient File. Government of Canada. https://food-nutrition.canada.ca/cnf-fce/index-eng.jsp (January 18, 2018).

3. Goucher, L.; Bruce, R.; Cameron, D.D.; Lenny Koh, S.C.; Horton, P. The environmental impact of fertilizer embodied in a wheat-to-bread supply chain. *Nature Plants* **2017**, *3*, 17012.

4. Guidice, A.L.; Clasadonte, M.T.; Matarazzo, A. LCI Preliminary Results in the Sicilian Durum Wheat Pasta Chain Production. *J. Commodity Sci. Technol. Quality* **2011**, *50*, 65 - 79.

5. Maskus, H. *Pulse milling project year 4: Summary of results*; Canadian International Grains Institute: Winnipeg, Manitoba, Canada, 2014.

6. Blonk Consultants. Agri-footprint 2.0. Part 2: Description of data. Blonk Consultants. https://simapro.com/wp-content/uploads/2016/03/Agri-footprint-2.0-Part-2-Description-of-data.pdf (January 18, 2017).

7. Jeswani, H.K.; Burkinshaw, R.; Azapagic, A. Environmental sustainability issues in the food–energy–water nexus: Breakfast cereals and snacks. *Sustainable Production and Consumption* **2015**, *2*, 17-28.

8. MacWilliam, S.; Parker, D.; Tremorin, D.; Marinangeli, C.P.F. Systematic Review and Meta-analysis of the Greenhouse Gas Emissions from using Dry Peas (Pisum sativum L.) and Lentils (Lens culinaris M.) in Crop Rotations in Western Canada. Saskatchewan Research Council. https://www.src.sk.ca/white-paper-carbon-emissions-pulse-crops (March 27, 2018).

9. Hegger, S.; Hischier, R. *Ecoinvent Version 2.2: Assessment of Assignment of Characterisation Factors to the Swiss LCI database ecoinvent (version v2.2)*; Swiss Centre for Life Cycle Inventories: Zurich, Switzerland, 2010.

10. Koch, P.; Salou, T. *Agribalyse: Rapport methodology. Version 1.3*; ADEME: The French Environment and Energy Management Agency: Angers, France, 2016.

11. Itten, R.; Frischknecht, R.; Stucki, M. *Life Cycle Inventories of Electricity Mixes and Grid. Version 1.3*; Treeze Ltd.: Zurich, Switzerland, 2012.

12. Government of Canada. Table of Reference Amounts for Food. Government of Canada. https://www.canada.ca/en/health-canada/services/technical-documents-labelling-requirements/table-reference-amounts-food.html (January 18, 2018).
